# Deadliest Animals with the Thinnest Wings: Near-Infrared Properties of Tropical Mosquitoes

**DOI:** 10.1177/00037028251341317

**Published:** 2025-06-12

**Authors:** Meng Li, Hampus Månefjord, Julio Hernandez, Lauro Müller, Christian Brackmann, Aboma Merdasa, Carsten Kirkeby, Mengistu Dawit Bulo, Rickard Ignell, Mikkel Brydegaard

**Affiliations:** 1 Department of Physics, 5193 Lund University, Sölvegatan 14C, 22362 Lund, Sweden; 2 Norsk Elektro Optikk, Østensjøveien 34, 0667 Oslo, Norway; 3 Department of Biology, 5193 Lund University, Sölvegatan 37, 22362 Lund, Sweden; 4 Dept. Biology, Dept. Ophthalmology, 5193 Lund University, Lund, Sweden; 5 Dept. of Veterinary and Animal Sciences, University of Copenhagen, Copenhagen, Denmark; 6 Dept. Plant Protection Biology, Swedish Agricultural University, Alnarp, Sweden

**Keywords:** Mosquito, biophotonics, thin-film, tissue spectroscopy, ballistic light, small animal imaging, hyperspectral, lidar, remote microscopy

## Abstract

Tropical mosquitoes transmit diseases like malaria, yellow fever, and Zika. Classifying mosquitoes by species, sex, age, and gravidity offers vital insights for assessing transmission risk and effective mitigations. Photonic monitoring for mosquito classification can be used in distributed sensors or lidars on longer ranges. However, a reflectance model and its parameters are lacking in the current literature. This study investigates mosquitoes of different species, sexes, age groups, and gravidity states, and reports metric pathlengths of wing chitin, body melanin, and water. We use hyperspectral push-broom imaging and laser multiplexing with a rotation stage to measure near-infrared spectra from different angles and develop simple models for spectral reflectance, including wing thickness and equivalent absorption path lengths for melanin and water. We demonstrate wing thickness of 174 (±1) nm – the thinnest wings reported to our knowledge. Water and melanin pathlengths are determined with ∼10 µm precision, and spectral models achieve adjusted R² values exceeding 95%. While mosquito aspect angle impacts the optical cross-section, it alters shortwave infrared spectra minimally (∼2%). These results demonstrate the potential for remote retrieval of micro- and nanoscopic mosquito features using spectral sensors and lidars irrespective of insect body orientation. Improved specificity of vector monitoring can be foreseen.

## Graphical abstract

**Graphical abstract:**
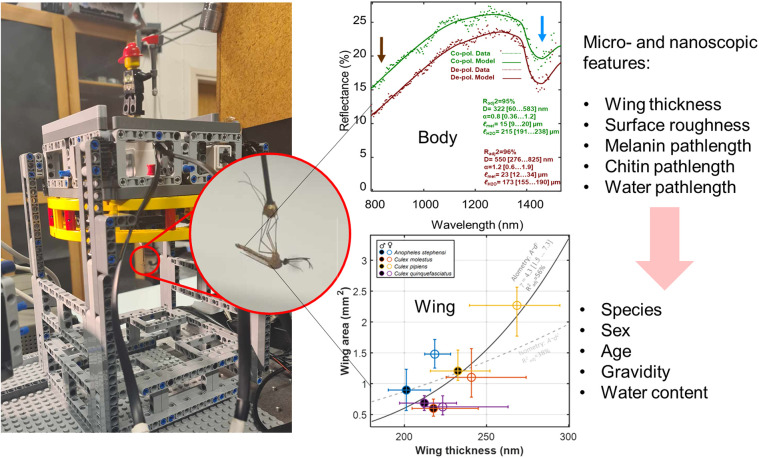
This is a visual representation of the abstract.

## Introduction

Mosquitoes are the deadliest animals on our planet due to their role in transmitting vector-borne diseases.^1^ Human diseases, such as malaria, dengue, yellow fever, and Zika, as well as livestock diseases, including West Nile fever, bluetongue, and the Schmallenberg virus (transmitted by midges), have significant impacts globally. Many of these diseases affect rural populations in tropical regions with poor housing quality,^2,3^ leading to reduced life expectancy, higher stillbirth rates, poverty, and hindrance to development.^4^ Outbreaks also occur in North America following the socioeconomic crisis,^5,6^ and global warming is expanding the range of vectors and pathogens into northern Europe.^7^

Despite continuous public monitoring and reporting of environmental risk factors, such as ozone, NO_2_, SO_2_, air quality,^8^ and birch pollen,^9^ the monitoring of deadly vectors lags behind.^10,11^ Mosquito populations can fluctuate dramatically over seasons,^2^ and strategically timed elimination can locally eradicate transmission for years.^13^ Accurate species identification is crucial for risk assessment, as not all mosquitoes pose a threat to humans. Many species do not blood-feed on humans and only older, previously blood-fed females transmit diseases. Moreover, sub-species can have distinct feeding timings and preferences.^14,15^ For example, the size of *Aedes aegypti* correlates with blood meals from multiple hosts, increasing infection risk.^16^ Therefore, effective public monitoring of vector-borne disease risk requires identification of mosquito species, sex, size, and life stage.

Wingbeat frequency (WBF) is a key feature for monitoring mosquitoes in flight. Mosquitoes can be distinguished from other insects by their high WBF, a characteristic partly linked to acoustic sexual attraction for mating in darkness.^17^ Various studies have proposed mosquito sensing by WBF, including acoustic sensing via smartphones in bedrooms,^18,19^ optical sensors using transmittance,^20^ or backscatter.^21,22^ These methods have shown success in classifying a limited set of species and sexes in controlled,^20–22^ and semi-wild environments.^23^ However, in situ identification by WBF alone is impractical. Males have significantly higher WBFs than females, but WBF can overlap between sexes of co-existing species. Additionally, WBF increases with gravidity,^24^ and air temperature.^25^ Although some species exhibit statistically different WBFs,^26^ the intra-species variation is about 25% even under constant conditions. This variability limits the differentiation of species based solely on WBF, especially in habitats with multiple mosquito species.

Harmonic overtone analysis of time series signals,^27,28^ and spectral analysis of signals from the wings and bodies of insects,^29–31^ have been shown to improve species specificity beyond WBFs. However, the physical mechanisms behind these improvements, the origin of harmonic overtones, and the factors governing reflectance from mosquito bodies remain unclear. This study aims to bridge these gaps by providing physical models for optical scattering by wings and bodies from several species of tropical disease vectors, including variations in sex, age, and gravidity. We demonstrate that microscopic and nanoscopic pathlengths of melanin, water, and chitin can be extracted from optical signals. We showcase spectral variations depending on the aspect angles of mosquitoes. Additionally, we provide the first quantitative values for the parameters governing the spectral reflectance of mosquitoes and show that these parameters significantly differ among species, sex, age, and gravidity.

###  Experimental

The study of optical backscattering signatures, specifically through the wavelength and angle-dependent backscatter (σ_*B* *S*_) and extinction (σ_*E* *x* *t*_) cross-sections, offers a powerful method for the optical sensing of mosquitoes. Dedicated optical systems, such as entomological lidars,^32^ the electronic backscatter optical scanning system (EBOSS),^33^ and various e-trap systems,^34,35^ use single-band,^36^ or multiband^24,37^ illumination and detection to study the interactions of light with mosquitoes. These systems enable detailed analysis of species composition and activity patterns.

The backscatter cross-section (σ_*B* *S*_) quantifies the amount of light reflected towards the sensor. It depends on the mosquito's projected area *A*(ψ, ϕ), which varies with its orientation (yaw and pitch angles, ψ and ϕ, relative to the source and detector), and its reflectance *R*(λ), the wavelength-dependent of reflected incident light. The extinction cross-section (σ_*E* *x* *t*_) provides a complementary perspective by measuring the total attenuation of light due to both scattering and absorption as it passes through a mosquito. This attenuation depends on the mosquito's size, shape, and tissue composition, through its projected area, *A*(ψ, ϕ), and its wavelength-dependent optical properties (λ). The σ_*E* *x* *t*_ offers insights into a mosquito's interaction with light beyond direct backscatter, making it valuable in environments where backscatter signals might be weak or difficult to isolate.

To gain even greater insights, both σ_*B* *S*_ and σ_*E* *x* *t*_ can be decomposed into envelope (σ_*body*_) and oscillatory (σ_*wing*_) components.^32,38^ The envelope component reflects the mosquito path through the beam profile and the body size, while the oscillatory components capture the wing dynamics and their bidirectional reflectance distribution function (BRDF). This distinction is key for identifying species-specific characteristics and flight behaviors. To increase measurement precision, techniques, such as sliding min/max windows adjusted to wingbeat frequency (WBF), have been developed.^31,32,39^ These methods separate the scattering contribution from the wings and body, enabling differentiation of species by wing interference,^39,40^ or deducing information from the abdomen, such as gravidity,^24^ biomass,^41^ or pathogen content.^42^

While a simplified model approximates the mosquito body as a volumetric ellipsoid and the wings as flat ellipses, the intricate microstructures of mosquito anatomy introduce complexities to real-world scatter. Importantly, the scattered light from the elliptical mosquito body exhibits omnidirectional characteristics resembling the Henyey–Greenstein scattering phase function dominated by forward scatter.^43^ In contrast, the flat wings scatter light directionally, similar to falling ice flakes.^44,45^ This specular property, in combination with wing dynamics, produces flashes,^46^ and harmonic overtones, which can differentiate closely related mosquito subspecies.^21^ A summary of these contrasting anatomical and optical characteristics is presented in Table I. This differentiation is crucial for the development of optical mosquito sensing systems.

**Table I.** Optical properties and physical characteristics of mosquito anatomy. This is a concise overview highlighting the distinct anatomical features of mosquito bodies and wings, their spatial and temporal properties, and the underlying optical and physical mechanisms.

Anatomy	Spatial shape (μm)	Temporal (μm)	Frequency (kHz)	Goniometric (°)	Polarimetric (% DOLP^a^)	Spectral (nm)	Physics
Body	Ellipsoid	Envelope	Low-pass convolution	Henyey–Greenstein, Omnidirectional	Partial co-polarized	Absorption by melanin and H_2_0	Ballistic tissue transport
Wings	Two elliptical planes	Waveforms and flashes	Wingbeats harmonics	Specular, directional	Co-polarized	Interference in chitin	Thin film

^a^ DOLP: Degree of linear polarization.

To characterize mosquito near-infrared optical properties using wavelength and angle-dependent analysis, we conducted three complementary experiments. These experiments independently investigated key physical features relevant to mosquito classification. The following sections detail these experiments. First, the wing reflectance experiment used a hyperspectral camera to capture specular reflectance from mosquito wings. Its primary objective was to quantify nanometric differences in wing membrane thickness and assess how these structural variations influence species- and sex-specific spectral reflectance signatures. Second, the body reflectance experiment, also using a hyperspectral camera, captured reflectance signals from live mosquito bodies. This aimed to extract information about their internal composition, specifically focusing on water and melanin content. By using polarized and depolarized hyperspectral imaging, we quantified diffuse reflectance from mosquitoes of different species, sexes, and ages. Lastly, the aspect angle dependence experiment, with a goniometry system (BIOSPACE), assessed how the orientation of mosquitoes relative to light source and detector would affect their backscatter signature across multiple wavelengths. This aspect-angle dependence is essential for accurately interpreting optical mosquito monitoring data collected remotely in natural settings.

### Wing Reflectance: Specular Wing Scattering Provides Species Contrast Due to Nano-Features

The BRDF for clear mosquito wings is close to specular, resulting in flashes when the surface normal of the wings coincides with the light source-detector midpoint. The two wings have four surface normals, which when convoluted by the wing flatness and illumination and detection aperture covers a large fraction of the unit sphere, creating a high likelihood of a specular flash for each wingbeat of the mosquito. The dynamical wing orientation can be accurately described by just three harmonics.^47,48^ Consequently, the geometrical cross-section of mosquitoes can also be described by three harmonics.

Mosquito wingbeats have a period of 1–3 ms, during which the observed flashes of backscattered light can contain at least 30 overtones.^46^ The presence of large number of overtones in the optical signals is related to the strongly directional scattering of mosquito wings, which behave neither as perfect mirrors nor as Lambertian (omnidirectional). This directional scattering can be approximated *I*(θ) = *I*_0_·cos(θ)^1/*r*^, where *I*_0_ is the incident intensity, θ is the scattering angle, and surface roughness *r* ranges from 0 to 1, with 0 representing an ideal mirror and 1 an ideal diffuser. Due to this highly directional scattering, even small periodic variations in wing orientation, captured by only three harmonics, translate into sharp, repetitive intensity peaks. These peaks result as numerous overtones in the backscattered optical signals. These flashes are commonly observed and have been previously extracted as signal features.^21,28^ The flashes typically constitute the largest fraction of the total light scattering by insects,^38^ and can be retrieved over distances of at least a hundred meters by entomological lidars.^40,46^ The flash duration, limited by the wing flatness and numerical aperture of the source and detector, can be as short as 130 μs.^49^ The flash is particularly pronounced for wavelengths matching the criterion for resonant back-scattering according to the thin-film interference between reflection from the front and back surface of the chitin wing membrane (Eq. 1).^63^

(1) $$\begin{array}{cc} \lambda_{\mathrm{Rmax}}=\frac{2dn_{chi}}{m-\frac{1}{2}}\sqrt{1-\frac{\mathrm{si}n^{2}\theta }{n_{\mathrm{chi}}^{2}}}, & m\in N \end{array}$$

in which λ_Rmax_ are wavelengths with resonant backscatter, *d* is the thickness of the wing, *n*_chi_ is the refractive index of chitin, θ is the light incidence angle, and *m* is the mode number. For backscattering instruments such as lidar, θ is zero and the square root term can be omitted.

Numerous studies of wing interference patterns (WIPs) have indicated great species contrast and sexual dimorphism. Studies include large sample sizes,^50–56^ surveyed by simple RGB imagers, which cannot quantitatively determine membrane thickness. Spectral case studies,^46,57^ describe the relation to the membrane thickness. Non-imaging studies in flight chambers,^21^ or with lidar,^40^ indicate that spectral flash properties, and thus effective thickness differ between closely related subspecies,^21^ which cannot be differentiated without genetics. Hyperspectral imaging of insect wings,^30,58–60^ can provide quantitative thickness maps of wing membranes. It is conceivable that such quantitative hyperspectral images, capturing both wing shape,^61^ vein structure, melanization, and membrane thickness, provide enough quantitative information to classify each individual to species and even subspecies level.

### Materials and Methods

**Figure 1.**
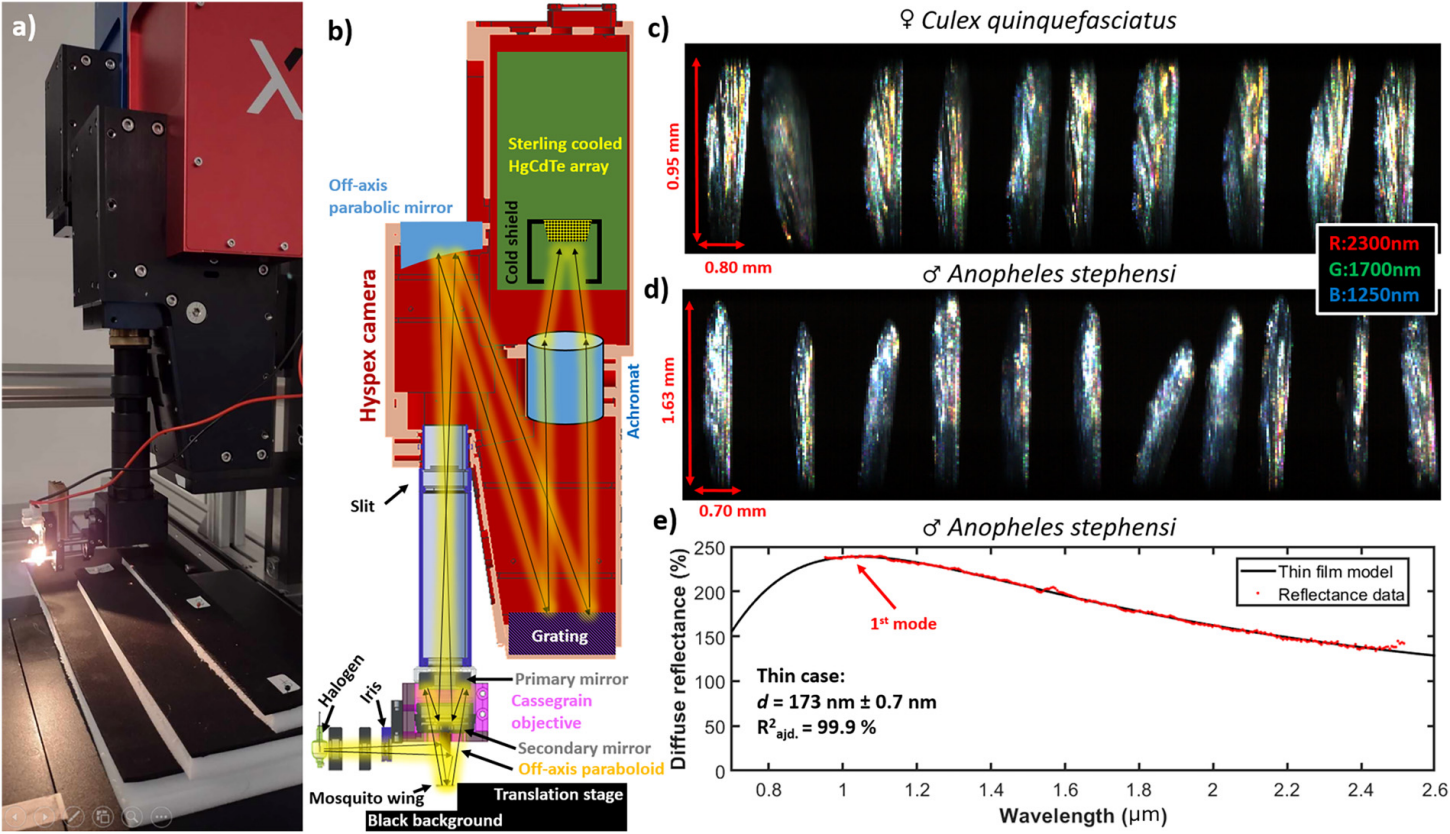
Hyperspectral imaging of mosquito wings. (a) Picture of the setup at Norsk Elektro Optikk, Oslo, Norway. (b) Approximate ray diagram for the setup, including the custom-made objective made for the purpose. (c,d) False-color short-wave infrared images of mosquito wings. (e) An example of fitting the thin film model to measured reflectance data. In this case, the first interference mode is on the edge of the spectral range of the instrument.

Mosquito specimens were reared at the Swedish University of Agricultural Sciences (SLU), Alnarp, Sweden.^21,44,62–64^ A total of 428 wings from dead male and female mosquitoes from four species: *Anopheles stephensi* (110 wings), *Culex pipiens Biotype Molestus* (hereafter *Cx. p. molestus*; 106 wings), *Culex pipiens biotype Pipiens* (hereafter *Cx. p. pipiens*; 110 wings), and *Culex quinquefasciatus* (102 wings) were detached and glued on a black edge of neoprene foam using a stereo microscope. The wings were mounted 20 mm above a black neoprene background, see Figure 1a. The mounted wings were brought to Norsk Elektro Optikk’s laboratories in Oslo, Norway, for hyperspectral imaging.

To obtain adequate magnification, a custom microscope objective was three-dimensionally (3D) printed (Figure 1b). The setup includes a 10 W halogen–tungsten filament, which was imaged onto the object plane by a 90° off-axis parabolic gold mirror. The backscattered light was collected by a reflective gold objective (Edmund Optics), which imaged the wings onto the slit of a hyperspectral camera (Hyspex SWIR, Norsk Elektro Optikk). The off-axis paraboloid and reflective objective were in a coaxial configuration. Spatial calibration was done by imaging a ruler. The hyperspectral camera provided 288 bands covering 900–2500 nm. Second-order diffractions were rejected by a coating on the HgCdTe imager.

Hyperspectral images were calibrated to diffuse reflectance by a grey standard. Multiple scans with different focuses were made, and the sharpest wing was cropped out by a custom Matlab script (The MathWorks, USA). The area of the wings was extracted from the masks. Each wing image was averaged spatially, and reflectance spectra, *R*_wing_(λ), were calculated. The measured reflectance spectra were fitted to a thin-film model *Ȓ_win_*_g(λ)_ (Figure 1e), with Eq. 2 representing an updated model based on previous thin-film work.^30,40,59,63^ Here, *F*_(λ,d)_ is the fringy reflectance from a thin film at wavelength and thickness *d.*^63^

(2) $$\begin{array}{rl} & F_{\left(\lambda ,d\right)}=\frac{4R_{\mathrm{Fresnel}}\mathrm{si}n^{2}\left(\frac{2\pi dn_{\mathrm{chi}}}{\lambda }\right)}{{\left(1-R_{\mathrm{Fresnel}}\right)}^{2}+4R_{\mathrm{Fresnel}}\mathrm{si}n^{2}\left(\frac{2\pi dn_{\mathrm{chi}}}{\lambda }\right)},\\ & R_{\mathrm{Fresnel}}={\left(\frac{n_{\mathrm{air}}-n_{\mathrm{chi}}}{n_{\mathrm{air}}+n_{\mathrm{chi}}}\right)}^{2},n_{\mathrm{chi}}=1.517+\frac{8800nm^{2}}{\lambda^{2}}\\ & {\hat{R}}_{\mathrm{wing}\left(\lambda \right)}=\frac{aF\left(\lambda ,d\right)\lambda^{k}+b\lambda_{0}^{k}}{\lambda_{0}^{k}+\lambda^{k}} \end{array}$$

in which the variable λ is the wavelength of light that interacts with the mosquito wing, and *d* is the wing's chitin layer thickness. *R*_Fresnel_ is the reflectance based on the refractive indexes of air, *n*_air_, and chitin, *n*_chi_;^68^ *n*_air_ is almost always considered as 1, and *n*_chi_ changes with λ. The coefficients *a* and *b* are used to adjust the model. The exponent *k* influences how reflectance changes with wavelength.

In a previous study, the spatial thickness heterogeneity was evaluated over each wing by letting fringe bias and amplitude depend on a cut wavelength, λ_0_, as short- and long-pass functions, respectively.^30^ This is not possible in this study, because we only observe a single fringe period within the spectral range of the short-wave infrared instrument due to thinner wings than previously reported. Consequently, k is set to zero and λ_0_ is indeterminable. To resolve more fringes, future hyperspectral imaging of mosquito wings should be carried out by instruments based on conventional Si CMOS image sensors in the visible to near-infrared regime. These image sensors also feature better resolution and reduced cost.

## Results and Discussion

The wing reflectance model explained 97% [94…99%] (median *R*^2^_adj_. and IQR respectively) of the measured reflectance yielded wing thickness confidence intervals (CI) of ±1.0 nm or ±0.4% relative precision.

The reflectance measurements indicate that the first interference mode for resonant backscattering is captured within the shortwave infrared range. Consequently, the spectral modulation depth is extremely high with most of the specimens exceeding 90%. This implies that photonic sensors,^24,64,65^ and lidars,^32,66,67^ retrieving backscattered wingbeat waveforms from mosquitoes, will be extremely sensitive to the choice of wavelength in relation to the wing thicknesses. This is the case for single-band systems,^32^ whereas dual-band,^37^ or hyperspectral systems,^40^ would have an increased likelihood of capturing flashes and harmonics in one or another band.

**Figure 2.**
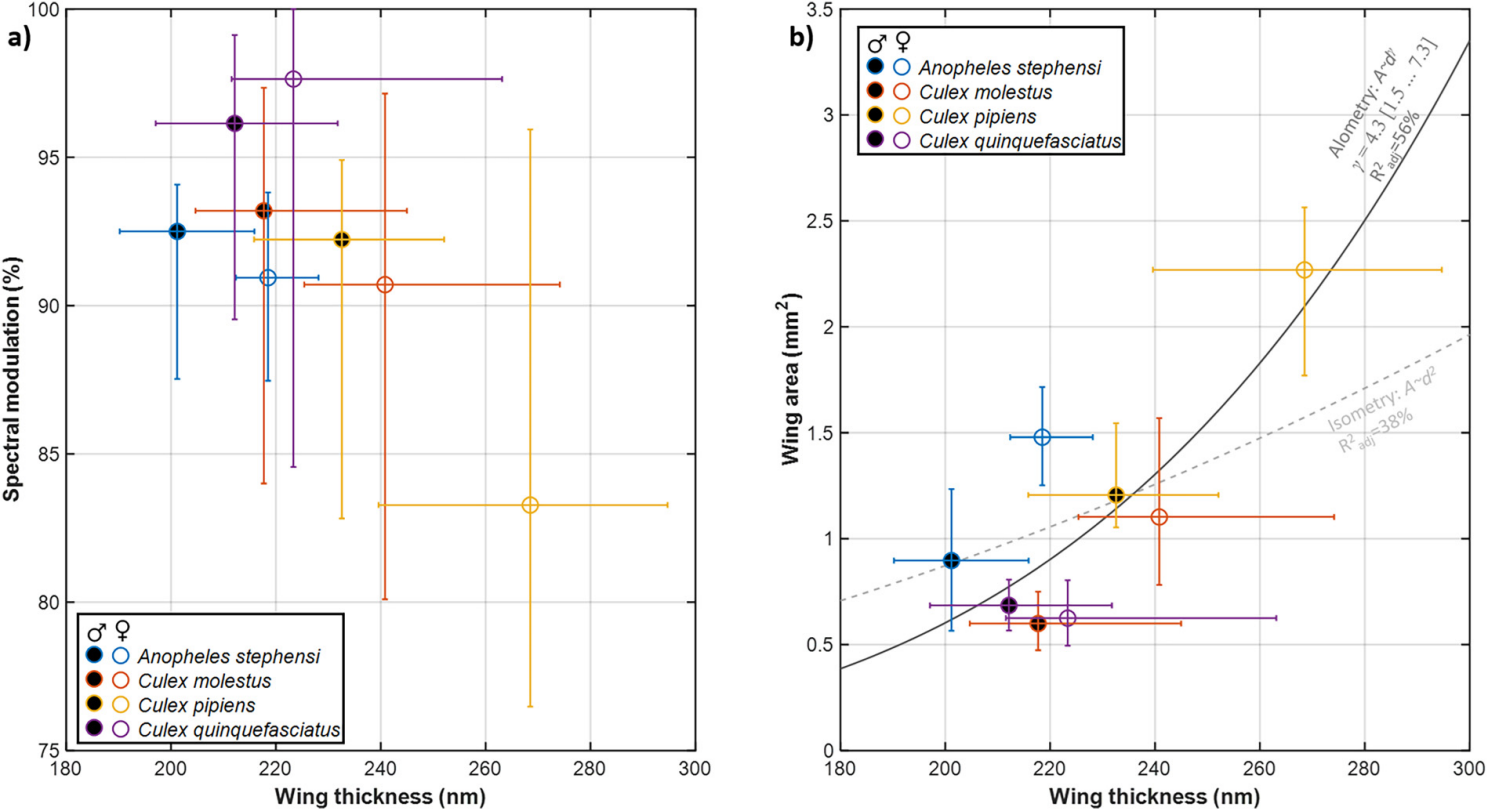
Measured specular properties of mosquito wings. (a) Median overall wing thickness for the investigated species of both males and females. The bars indicate within-species and sex spread by IQR. The spectral modulation depth, a/(a + b) from Eq. 2, is very high, exceeding 90% for most cases. (b) Wing membrane thickness correlates weakly with the area.

The effective wing thickness ranges from ∼200 to ∼300 nm, see Figure 2a. To our knowledge, these are the thinnest wings reported in literature. A thickness of 358 nm was previously reported from a much larger mosquito species.^68^ Insect wings are always thinner on the hind edge,^30,40^ thus wings are locally thinner than the effective thicknesses in Figure 2. Chitin polymer chains are spaced by 1 nm, but the hierarchical preferences form nanofibrils of 3 nm and larger nanofibers with 20 nm spacing.^69^ Therefore, mosquito wings are quite close to the quantized world of molecular sizes. Quantum entomology, in which wing membranes vary discretely is thus possible:

- Specificity: The intraspecies and intrasexual variations were estimated by interquartile ranges (IQRs) indicated in Figure 2. The spread was as small as ±10 nm or ± 5% for *An. stephensi*. Although many of the groups overlap there are also cases with significant discrepancies in wing thickness between sexes, e.g., *An. stephensi*, and between species, e.g., *An. stephensi* versus *Cx. p. pipiens*.
- Allometry: The wing thickness of the investigated species only correlated with the wing area by 56% across species and sexes (see Figure 2b) and the allometric relation exponent, γ, is uncertain and isometry cannot be excluded (γ = 2). Previous studies showed that wing thickness and area can deviate significantly from isometry.^30,40,46,59^

**Figure 3.**
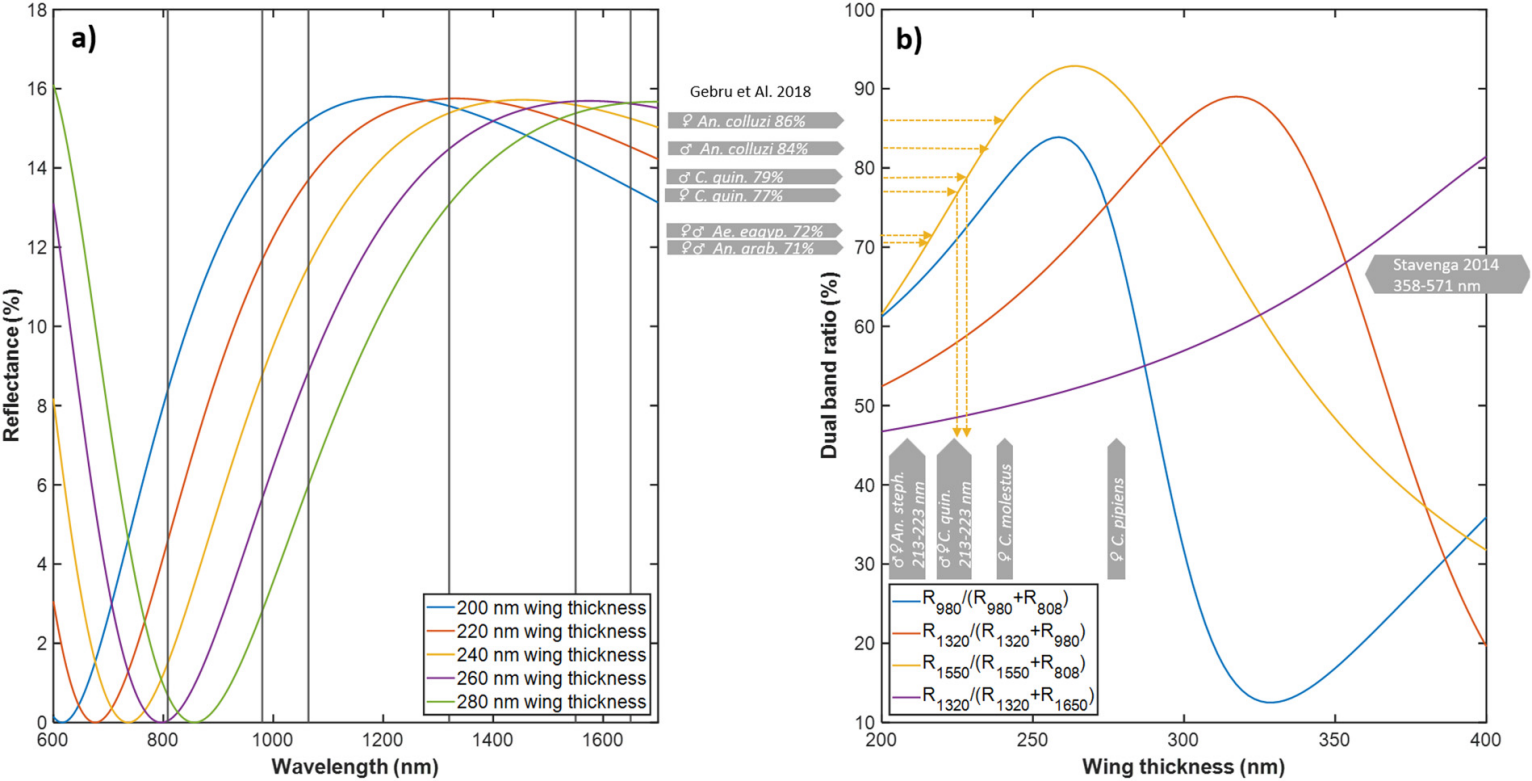
Effect of observed range of wing thicknesses on specular reflectance responsible for wing beat flashes. (a) Modelled reflectance of the thickness spans observed in this study in the near- and short-wave infrared range. The black lines indicate common laser bands previously used in entomological sensing. (b) Dual-band ratios modeled between common laser bands. Our current recordings match ratios from a previous study within 10 nm.

We compared observed mosquito wing membrane thickness ranges against common laser wavelengths, previously used in entomological sensors,^22,31,70^ and lidars^24^ (Figure 3). In particular, near-infrared (NIR) bands (808–980 nm) display great sensitivity to the wing membrane thickness. Several dual-band sensors and lidars have been reported, Figure 3b, to display the dual-band ratio for specular wing beat flashes as a function of membrane thickness. In one case, we can compare this ratio to 10-year-old recordings from our laboratory.^21^ At that time, the thicknesses could not be uniquely determined but it was pointed out as a means to distinguish between related species, which are almost impossible to differentiate by microscopy by taxonomists. We can here verify that *Cx. quinquefasciatus* wing membranes are 213–223 nm, with a discrepancy of just 10 nm to the previous study.^21^ Additionally, both studies show that female wings are generally thicker than male wings

### Body Reflectance: Quantitative Microscopic Features Retrieved from Diffuse Body Reflectance

Light transport in scattering media, such as biological tissue, is governed by absorption and reduced scattering coefficients, μ_a_ and μʹ_s_, in metric units.^71^ Light diffusion^72^ and photon migration^73^ theories were developed on macro scales of centimeters for biomedical,^74^ and life science applications.^75^ Mosquitoes are over a thousand times smaller than humans, but μ_a_ for water and melanin remain constant. Although the highest μʹ_s_ is reported in insects,^76^ the scattering mean-free-path is bound by the wavelength of light. Consequently, near-infrared light interaction with mosquitoes is ballistic, similar to X-ray imaging of humans, or optical clearing scenarios,^77,78^ where scattering is low compared to the organism sizes.^79^

As opposed to diffuse light transport, ballistic interrogation implies that most photons inside mosquitoes keep their initial propagation direction, polarization state, and phase. It also implies that the interrogation pathlength of backscattered light is longer than the pathlength for transmitted or forward-scattered light.^43^ Consequently, backscatter can display larger depolarization and equivalent water absorption pathlength than transmitted light. Melanized cuticles are mainly transparent for short-wave infrared light (λ > 1μm), and thus this light interrogates the entire body of mosquitoes. This has multiple applications for detailed mosquito diagnostics,^42^ for example, classification of species,^29^ and sexes,^21^ estimation of age,^80^ detection of gravidity by depolarization,^24^ or even detection of pathogens by the tissue-spectral scatter coefficients.^81^

*Materials and Methods. *Several species of mosquitoes reared at SLU, including *Aedes aegypti*, *Anopheles coluzzii*, *Anopheles gambiae*, and *Culex quinquefasciatus*, were brought alive to a hyperspectral imager at the eye clinic at Skåne University Hospital. A total of 82 samples were scanned. These samples represented both sexes of multiple species, with ages ranging from 2 to 5 days post-adult emergence. The detailed species, age, and sex distribution among the 82 samples is summarized in Table II.

**Table II.** Summary of mosquito samples for body reflectance study (*n* = 82) by species, sex, and age.

Species	Sex	Age (days)	Number of samples
*Aedes aegypti*	Female	4	9
		5	3
	Male	4	10
		5	5
*Anopheles coluzzii*	Female	5	10
	Male	5	7
*Anopheles gambiae*	Female	4	6
	Male	4	7
		5	6
*Culex quinquefasciatus*	Female	2	6
		3	1
	Male	2	8
		3	4

**Figure 4.**
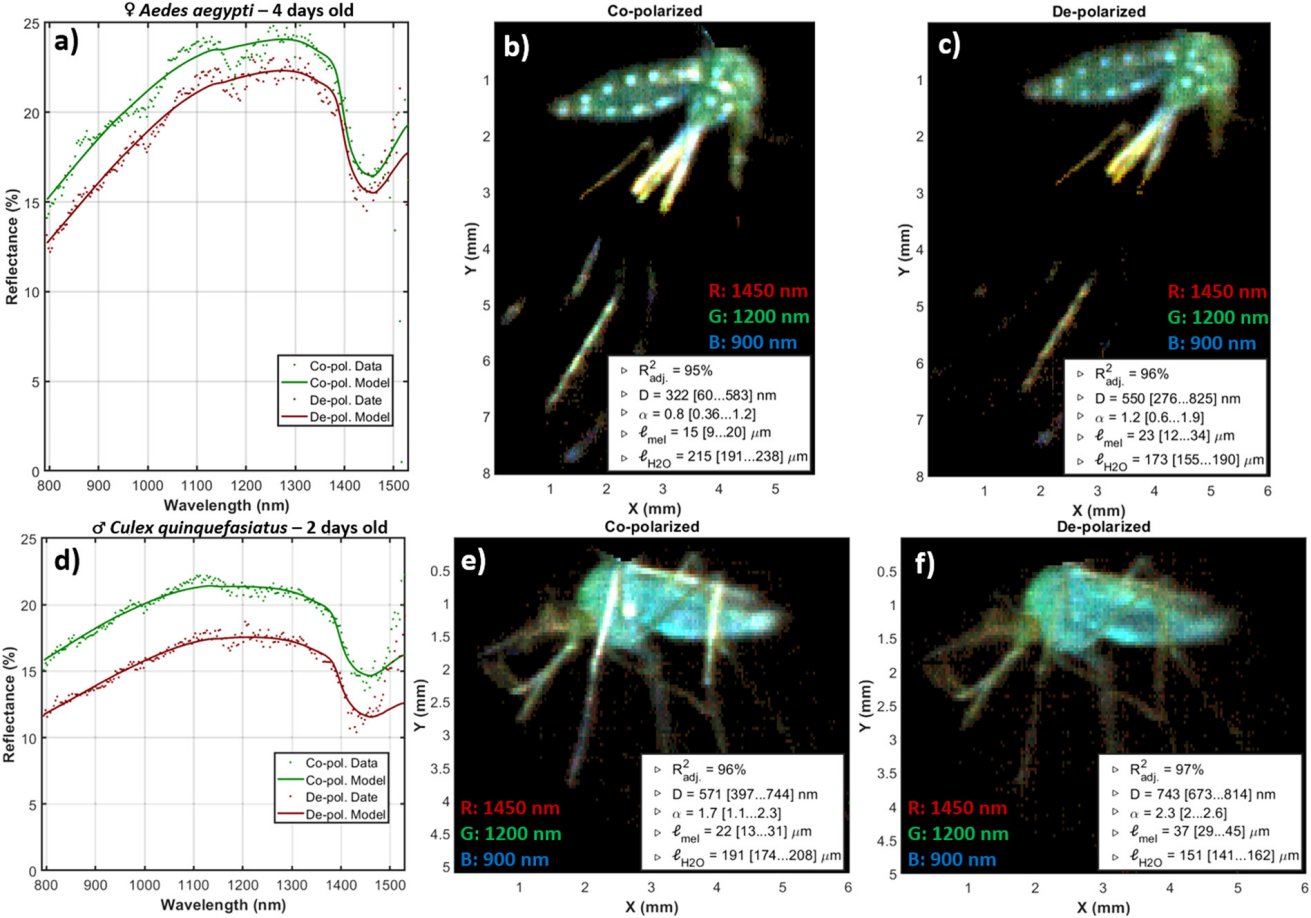
Hyperspectral short-wave infrared imaging of whole mosquitoes. (a,d) Co- and de-polarized reflectance for two mosquitoes. The spectra are offset by a flat specular contribution to the co-polarized image. (b,c,e,f) False-color infrared images of the same two mosquitoes. The blue color represents 900 nm and is diminished by melanin. The green color represents 1200 nm and is insensitive to melanin and water, whereas the red color represents 1450 nm and is diminished by water. The model parameters are indicated with model explanation grade and 95% confidence intervals. Note how the white spots on the *Aedes aegypti* diminish for de-polarized light, also note the absence of leg gloss in the depolarized image of the *Culex quinquefasciatus*.

The specimens were immobilized by rapid chilling and immediately scanned to avoid changes in the body structure after death and desiccation. Spectral imaging included whole mosquitoes with both body and wings towards a black background. The spectral imager (by Norsk Elektro Optikk),^82^ is based on a visible-extended indium gallium arsenide (InGaAs) camera using down conversion. For our experiment, the visible range (and thus their second-order refractions) was blocked by a RG780 long-pass filter. Hence, the usable spectral range was 382 spectral bands from 900 to 1600 nm. Two ultra-broadband wire grid linear polarizers (Meadowlark Optics, USA) were used to capture both co- and de-polarized images. The incidence angle of the light was 45°. The spectral imager was positioned at 0° with a working distance of 80 mm. The spatial resolution was 33 μm/pix. The hyperspectral image cube was white calibrated and flat field calibrated by a Lambertian gray standard, thereby obtaining an image calibrated to diffuse reflectance for both co- and de-polarized light. Each specimen was initially cropped out by hand from the hyperspectral image, and then a mask was automatically applied to exclude background pixels. The area of the mosquito was calculated by the number of pixels in the mask times their physical size in the object plane. The mean reflectance over the mosquito pixels was calculated for both polarizations, *R*_copol_(λ) and *R*_depol_(λ) (Figures 4a,d). The signal-to-noise ratio (SNR) of the average reflectance of this experimental setup was not great, approximately 50:1 in Figure 4a and 100:1 in Figure 4d. However, the SNR resembles what can currently be retrieved by hyperspectral lidar^40^ of free-flying insects in situ. This instrument lacks second-order rejection filters on the imager, thus the long end of the spectrum cannot be used.

Reflectance can be split into two parts,^83^ specular reflectance, *R*_spec_, and diffuse reflectance, *R*_diff_. Specular reflectance is often thought to be spectrally flat since it is caused by a mismatch in refractive index (*n*_1_–*n*_2_)^2^/(*n*_1_ + *n*_2_)^2^ since the refractive indices of tissues are simply given by the density.^71^ These statements are truths with modifications; biological interfaces are not step-changes from *n*_1_ to *n*_2_ but can have protruding subwavelength features causing gradient indices,^68^ also known as anti-reflectance features. For mosquito bodies, these features can comprise nanoscopic hairs or scales.^84^ In addition, the refractive index can deviate due to strong absorption bands, as described by the Kramers–Kronig relation. For mosquitoes, the near-infrared absorption is dominated by melanin and water, and their refractive indices do not change noteworthy in the near-infrared range. We estimated the wavelength-independent specular reflectance by the difference of co- and de-polarized reflectance across the spectral range of our instrument:

(3) $$R_{\mathrm{spec}.}=|R_{\mathrm{copol}.(\lambda )}-R_{\mathrm{depol}.(\lambda )}{|}_{\mathrm{median}}$$

There are multiple approaches for modeling light transportation in scattering media, and their usefulness depends entirely on the application. Our aim was to deduce microscopic metric features from the backscattering cross-section or spectral reflectance of free-flying mosquito bodies. The theory for diffuse reflectance that we found adequate for our purpose is the Kubelka–Munk theory.^85^ This was developed to estimate the reflectance from slabs or paper and paint in 1930s Czechia. Specifically, we adopt Eq. 4, which estimates diffuse reflectance by “thin specimens of poorly scattering material”.^85^ Kubelka–Munk state that the scattering and absorption mean-free-path, and the sample thickness, control the diffuse reflectance and transmittance of a sample, but since reflectance is dimensionless, metric factors must be relative to each other. Such metric coefficients are possible to quantify and compare between studies.

With knowledge about the spectral dependence of scattering and absorption, we can deduce metric features from reflectance in multiple spectral bands. We introduce the term “scatterance”, *S*(λ), and absorbance, *A*(λ). The spectral scatterance can in principle display complicated structures,^86^ but for the short-wave infrared region a simple power law suffices,^75,87^ for biological tissue. Diffuse reflectance, *R*_diff_, must converge to 100% when *S*→∞ and 0% when *A*→∞. Further, *R*_diff_=0 when *S* = 0. The following equation fulfills this:

(4) $$\begin{array}{rl} & {\hat{R}}_{body}\left(\lambda \right)=R_{spec.}+R_{diff\;\;}\left(\lambda \right)=R_{spec.}+\frac{S}{1+S+A}\\ & S\left(\lambda \right)={\left(\frac{D_{\frac{1}{2}}}{\lambda }\right)}^{\alpha },\;A\left(\lambda \right)=\ell_{H_{2}0}\mu_{H_{2}0}+\ell_{mel.}\mu_{mel.}\\ & {\hat{R}}_{b\mathrm{ody}}\left(\lambda \right)=R_{\mathrm{spec}.}+\frac{{\left(\frac{D_{\frac{1}{2}}}{\lambda }\right)}^{\alpha }}{1+{\left(\frac{D_{\frac{1}{2}}}{\lambda }\right)}^{\alpha }+\ell_{H_{2}0}\mu_{H_{2}0\left(\lambda \right)}+\ell_{\mathrm{mel}}\mu_{\mathrm{mel}\left(\lambda \right)}} \end{array}$$

in which *D*_½_ is the wavelength where half of the light is reflected without absorption or transmission. This parameter acts as a gain for diffuse reflectance. α, representing the spectral dependence of the scattering, is unitless, and can tilt the spectra, for example as a result of pathogens;^81^ ℓ_H2O_ and ℓ_mel_ are the equivalent absorption pathlengths in water and melanin, respectively; and μ_H2O_(λ) and μ_mel_(λ) are the absorption coefficients for pure water,^88^ and melanin.^48^ The coefficients, *D*_½_, α, ℓ_H2O,_ and ℓ_mel_ were fitted to the measured diffuse reflectance *R*_copol_–*R*_spec_ and *R*_depol_ by a numerical search algorithm (Curvefit Toolbox, Matlab).

**Figure 5.**
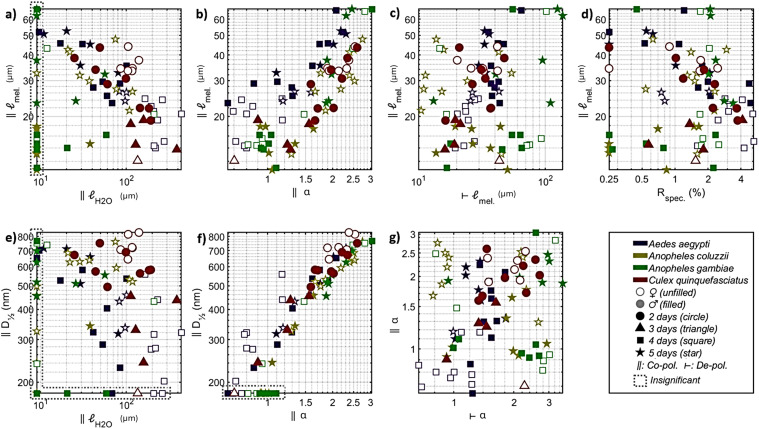
Mosquito body reflectance parameters among the investigated groups. (a) Co-polarized water versus melanin pathlength. Melanin pathlength anticorrelates with water pathlength. Females display stronger absorption than males. (b) Melanin pathlength versus spectral tilt, α. Despite a covariance, new groups are differentiated, see for instance male *Aedes aegypti* and female *Anopheles*. (c) The melanization can be entirely distinct for co- and de-polarized reflectance, presumably because of the micro-organization of the pigment. *Anopheles coluzzii* and *Anopheles gambiae* occupy opposite corners of this parameter space, even though they are closely related. (d) Despite the high refractive index of melanin, the specular reflectance varies independently since subwavelength structures and gradients have a greater impact. (e) The total tissue scatter does not increase with the water pathlength. (f) Spectral tilt, α, and total scatter, *D*_½_, covary but sexes protrude from the diagonal. (g) The spectra tilt differently for co- and de-polarized light, with the larger *Aedes aegypti* having the flattest spectra. The two *Anopheles* species display opposite spectral tilt for co- and de-polarized light, which is of interest as these highly related species are not possible to differentiate through microscopy.

*Results and Discussion. *The median specular reflectance was 3% (±1% IQR) for the 82 specimens. The model for diffuse reflectance achieved a median explanation grade, *R*^2^_adj_, of 96% (95…97% IQR). However, the lower confidence bound of all four coefficients, *D*_½_, α, ℓ_H2O_, and ℓ_mel_, was only distinct from zero in 46 cases of co-polarized reflectance, and 49 cases of de-polarized reflectance. Since the cases with insignificant coefficients showed strong preference for a specific group, we chose to include them on the axis of Figure 5, with a note.

The median water pathlength across all samples was 51 μm for co-polarized reflectance and 61 μm for de-polarized reflectance. The confidence was ±12 μm (±20% relative error) for co-polarized reflectance and ±13 μm (±17% relative error) for de-polarized reflectance. The equivalent water absorption pathlength only covaried slightly (21% *R*^2^_adj_.) with the area, ℓ_H2O_∼ *A*^γ^, in which γ = 1.3 (0.4…1.8 CI).

The median melanin pathlength across all samples was 25 μm for co-polarized reflectance and 32 μm for de-polarized reflectance. The confidence was ±11 μm (±27% relative error) for co-polarized reflectance and ±10 μm (±36% relative error) for de-polarized reflectance. The scattering parameter *D*_½_ was estimated with confidences of ±90 nm (±27% relative error). The scatterance, *S* ∼ *D*_½_α, scaled slightly (9% *R*^2^_adj_) with the area, *A*, with *D*_½_α ∼ *A*^γ^, in which γ=0.34 (0.23…0.44 CI). This gives a small indication that reflectance is contributed by the volume and depth.

We made a separate analysis of the white spots on *Ae. aegypti* in Figure 4bc. White color is rare in thin objects due to photon escape before multiple scattering.^76,84^ In addition, nanostructures with extreme scatter coefficients in the visible region have reduced scattering toward infrared when the structures are subwavelength. Indeed, the reflectance of the white spots on the *Ae. aegypti* abdomen could be explained by a short-pass function:

(5) $$R_{\mathrm{spot}(\lambda )}=\frac{1}{1+(\frac{\lambda }{D_{1/2}}{)}^{\alpha }}$$

where *D*_½_ is 1215 nm (1204…1226 nm CI) and α is 4.2 (3.9 … 4.5 CI). This confirms that the spots are white in the visible range below 600 nm, but steadily decrease in reflectance for longer wavelengths, supposedly since near-infrared wavelengths fail to resolve scattering nanostructures. It is noteworthy that previous studies,^76,84^ could not display such a decrease of reflectance towards infrared wavelengths.

### Aspect Angle Dependence: Measurement of Mosquito as Lidar Targets

Similar to radar cross sections,^89^ lidar cross-sections, σ_BS_, also depend on observational aspect angles of pitch and yaw. This poses both a challenge and an opportunity for assessing heading directions. The spherical geometrical cross-section of dried or immobilized insects could be calculated from 3D models of insects from photogrammetry.^43,47,89,90^ The wings can be detached from the body for independent measurements,^58,92^ since the body and wing scatter contributions can later be separated in the frequency domain for free-flying specimens. By combining kHz wing beat sensing and stereo vision,^21,31^ both body and wing cross-sections can be acquired simultaneously as the heading direction. Despite the complicated anatomy of insects, their elongated bodies can be approximated as an ellipsoidal spherical cross-section. Multiple symmetries (left/right, dorsal/ventral) apply to mosquito bodies, and measured cross sections at various aspects can be projected to a low number of polar or spherical harmonics.^43,58^ As it has been proposed that spectral or polarimetric band ratios could prove more stable for varying observation angles,^21,24^ this would be reasonable for the ballistic regime of small insects where the backscatter intensity is scaling with insect volume rather than cross-section.

*Materials and Methods. *We imaged six groups of mosquitoes, comprising male, as well as female virgin and gravid *An. coluzzii*. In addition, young and old *Cx. quinquefasciatus* females and males were imaged. Each group included two specimens, resulting in a total of 12 samples scanned.

**Figure 6.**
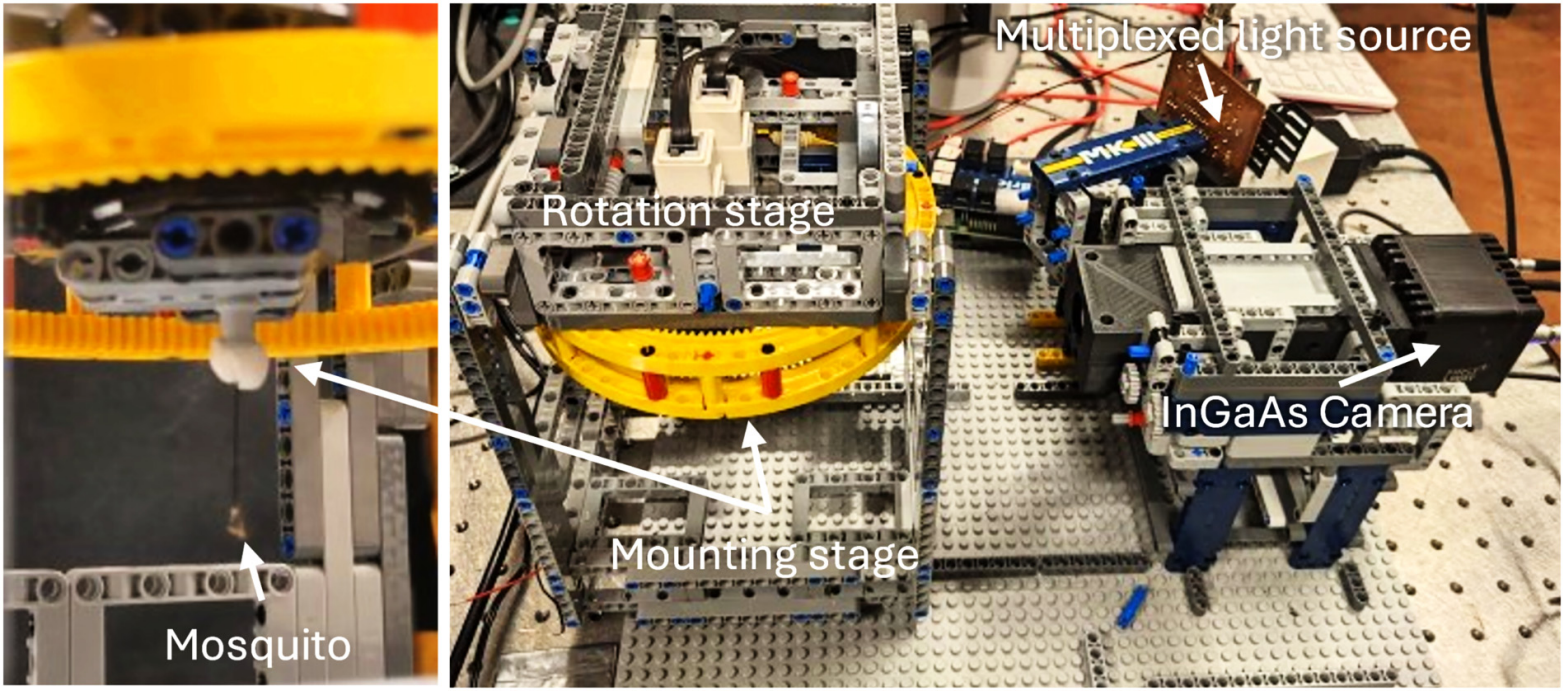
The instrumentation used for studying aspect angles of mosquito bodies with multispectral short-wave infrared images. An infrared version of the versatile BIOSPACE imaging.^93^

A multispectral multi-aspect imaging instrument was used for the analysis.^91,93^ The instrument was upgraded with an InGaAs camera (C-RED3, FirstLight, France), and laser diode multiplexing, to acquire multispectral images in seven shortwave infrared wavelengths spanning from 1000–1640 nm. The instrument has a rotation stage to rotate targets to various angles, analyzing different aspect angles, see Figure 6. Compared to previously,^91^ the target stage was flipped upside down for suspended mounting.

The wings were removed from freshly killed mosquitoes, following rapid chilling, and their bodies glued with their legs on pins. The pins were thereafter mounted onto the mounting stage of the instrument, with the mosquitoes hanging from the pins. The mosquito bodies were rotated 360° with 15° steps. For each aspect, a multispectral image was acquired. The images were flat-field and white calibrated by a diffuse grey reference. The precise laser diode emissions deviate from the specifications but were measured by an InGaAs spectrometer (Stellanet).

**Figure 7.**
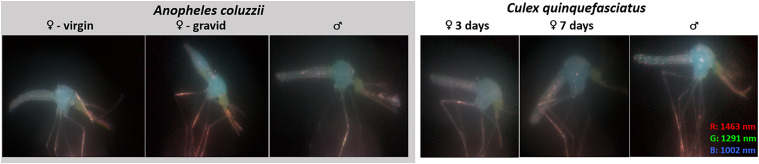
The six groups of mosquitoes visualized as false color infrared images. Anatomical features display distinct spectral contributions; the body primarily scatter short wavelengths (displayed in blue), since water attenuate the longer bands, the eyes display green tones since both melanin and water decrease eyeshine. Legs and antennae scatter long-wave light, and are seen as red.

*Results and Discussion. *The multispectral images revealed different spectral features of the mosquitoes. False-color images were formed by combining images of 1463 nm, 1291 nm, and 1002 nm as red, green, and blue, respectively (Figure 7). In the images, the bodies show a blue-cyan color, while the eyes appear green, and the limbs appear red.

Aspect angles: The yaw observation angle, ψ, foremost affects the cross-section regardless of the spectral band. The relation is adequately described by

(6) $$\hat{\sigma }(\lambda ,\psi )=k_{0}(\lambda )-k_{1}(\lambda )\cos\;(\psi )-k_{2}(\lambda )\cos\;(2\psi )$$

where σ is the backscatter cross-section as a function of wavelength and observation angle, ψ, (yaw). The head is in the direction where ψ = 0, *k_0_* is the average cross-section from all angles, *k_1_* is the reduction of the cross-section in the direction of the head, and *k_2_* is the increase of cross-section from the sagittal plane. Here, elongation is given by *k_2_/(k_2_* *+* *k_0_)* and the head/tail asymmetry is given by *k_1_/(k_1_* *+* *k_0_).* These dimensionless parameters are mainly constant across spectral bands, see Figure 9.

**Figure 8.**
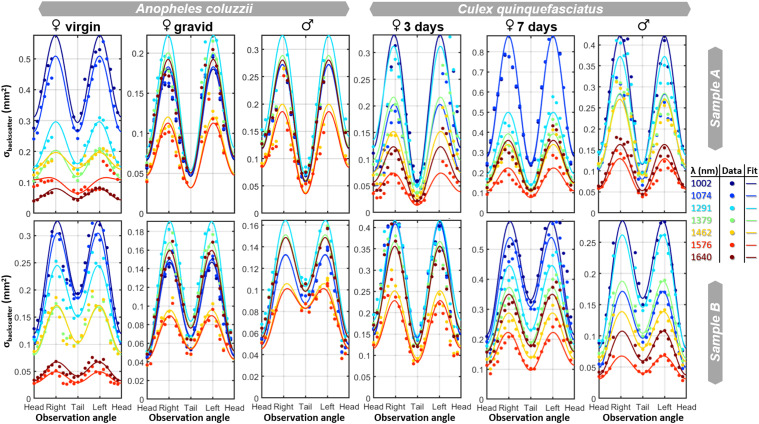
The measured cross section for two replicas from each of the six groups of mosquitoes, as a function of observation angle (yaw) and wavelength. The cross-section can be described by three polar harmonics. The spectral composition shows some dependence on observation angle and replicate, particularly between individual samples within the same species (e.g., male *An. coluzzii*). However, the spectral differences remain pronounced between the different mosquito groups.

We analyzed the spectral composition within the aspect scans in Figure 8 by singular value decomposition (SVD) and divided the eigenvalue by their sum to obtain relative variances. Across the specimens, the trend is that the first spectral component explains 91% of the spectra observed from all angles, the second component explains 5% of the variance, and the third component explains just 2%. The second and third spectral components could not be associated with melanin or water, rather the specular reflections from the legs and abdomen produce a strong glittering (Figure S1, Supplemental Material). Mosquitoes have relatively small eyes, and eye melanization could be more pronounced for other more visual vectors, such as horseflies.^94^ Differentiation of head and tail could also be more feasible with shorter bands, where melanin absorption is stronger. There are previous indications in the literature^21,95^ to indicate that spectral ratio has limited dependence on the observation angle. However, neither study or other studies have investigated spectral content in relation to all the aspect angles (yaw, pitch, and roll).

**Figure 9.**
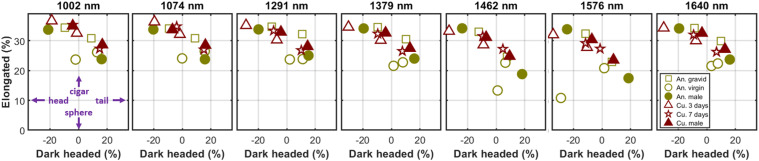
The elongation (*k*_2_/(*k*_2_ + *k*_0_)) and the head/tail asymmetry: dark headed (*k*_1_/(*k*_1_ + *k*_0_)) are shown for the 12 samples in all seven bands. The gravid anophelines display greater elongation than their virgin counterparts. The eye melanization shows surprisingly little reduction of the frontal cross-section. However, we note that *Culex* males and the older *Culex* females show a slightly dark frontal cross-section compared to the younger *Culex* females.

## Conclusion

This study presents measured near-infrared optical properties of mosquitoes with metric values and high precision, focusing on species, age, sex, and gravidity states. Our results demonstrate the thinnest (174 nm) insect wing in literature, to our knowledge, with a high precision (∼1 nm), as well as water and melanin pathlengths (∼10 μm precision). The spectral models derived from these measurements, with adjusted R² values over 95%, show a contrast between species, age, sex, and gravidity states of the mosquitoes. Moreover, our investigation reveals that, while the aspect angle of mosquitoes significantly influences their optical cross-section, the spectral variations in the shortwave infrared remain minimal (∼5%).

The methods have room for improvement; the spatial resolution of the hyperspectral imaging was marginal. We could extract some anatomical features, such as the spots on the yellow fever mosquito but could not provide a wing thickness map as reported in previous studies.^30,58^ A cheaper CMOS hyperspectral camera could be preferable to the infrared setup used in this study. The CMOS camera could capture more interference fringes in the visible regime compared to the infrared, which struggled to estimate thickness heterogeneity as previously achieved.^30^

The experimental VIS extended InGaAs hyperspectral camera only has a limited spectral range and suffers from second-order diffraction. The robustness of numerical fitting and the accuracy of the estimated reflectance parameters would greatly improve if reflectance could be measured on both sides of the 1450 nm water absorption band, and in particular if the 1940 nm water absorption bands could also be covered. The illumination of the fresh mosquitoes was suboptimal, and the SNR could have been better. On the other hand, the noisy reflectance spectra resemble those retrieved from free-flying insects by hyperspectral lidar.^40^

The ability to remotely retrieve micro- and nanoscopic features of mosquitoes using multispectral lidars presents a powerful tool for vector surveillance. By accurately distinguishing mosquito species, age, sex, and gravidity, targeted and efficient control strategies could be developed. This, in turn, could lead to improved management of mosquito-borne diseases, such as malaria, dengue, Zika, and others, directly enhancing global health outcomes. Further research could expand this work with more replicates and a wider range of mosquito species, incorporating additional variables, such as feeding status. Developing robust spectral libraries of mosquito optical properties would facilitate advanced classification algorithms for remote sensing applications. We acknowledge optical differences between lab-reared and wild mosquitoes may pose an issue, as studies show a need to retrain models.^96^ While current data is insufficient to quantify this, our physics-based model is potentially less sensitive to these variations compared to machine learning models. Ultimately, this research has laid a foundation for optical analysis to combat mosquito-borne diseases, which could in extension improve global health.

## Supplemental Material

sj-gif-1-asp-10.1177_00037028251341317 - Supplemental material for Deadliest Animals with the Thinnest Wings: Near-Infrared Properties of Tropical Mosquitoes Supplemental material, sj-gif-1-asp-10.1177_00037028251341317 for Deadliest Animals with the Thinnest Wings: Near-Infrared Properties of Tropical Mosquitoes by Meng Li, Hampus Månefjord, Julio Hernandez, Lauro Müller, Christian Brackmann, Aboma Merdasa, Carsten Kirkeby, Mengistu Dawit Bulo, Rickard Ignell and Mikkel Brydegaard in Applied Spectroscopy

sj-gif-2-asp-10.1177_00037028251341317 - Supplemental material for Deadliest Animals with the Thinnest Wings: Near-Infrared Properties of Tropical Mosquitoes Supplemental material, sj-gif-2-asp-10.1177_00037028251341317 for Deadliest Animals with the Thinnest Wings: Near-Infrared Properties of Tropical Mosquitoes by Meng Li, Hampus Månefjord, Julio Hernandez, Lauro Müller, Christian Brackmann, Aboma Merdasa, Carsten Kirkeby, Mengistu Dawit Bulo, Rickard Ignell and Mikkel Brydegaard in Applied Spectroscopy
