# Supplementary figures and images for: Deadliest Animals with the Thinnest Wings: Near-Infrared Properties of Tropical Mosquitoes
Source: Appl Spectrosc. 2025 Jun 12;79(11):1625–39. doi: 10.1177/00037028251341317 (PMC12569131; doi:10.1177/00037028251341317)

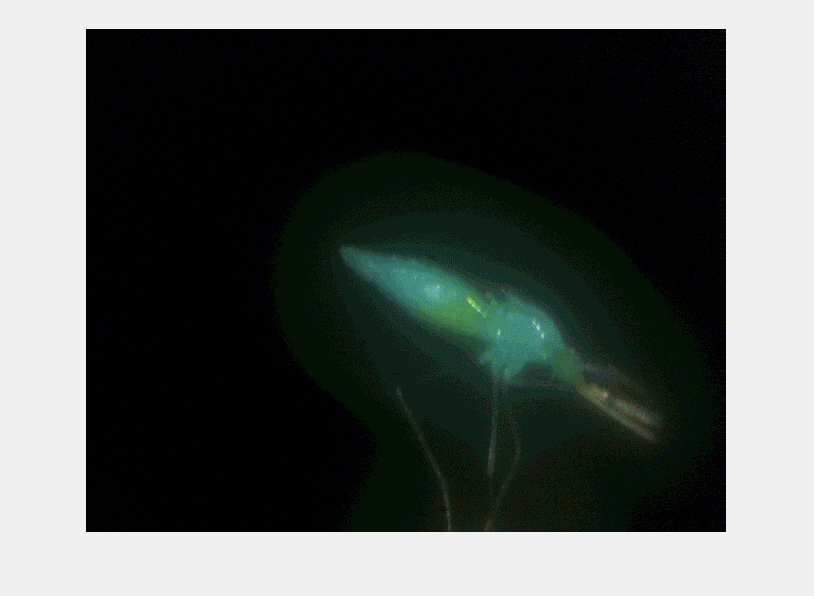

Supplement: sj-gif-1-asp-10.1177_00037028251341317 - Supplemental material for Deadliest Animals with the Thinnest Wings: Near-Infrared Properties of Tropical Mosquitoes [file sj-gif-1-asp-10.1177_00037028251341317.gif]
